# Marked Variations in Colon Cancer Epidemiology: Sex-specific and Race/Ethnicity-specific Disparities

**DOI:** 10.4021/gr2009.09.1311

**Published:** 2009-09-20

**Authors:** Robert John Wong

**Affiliations:** Department of Medicine, California Pacific Medical Center, 2351 Clay Street, Suite 380, San Francisco, CA USA 94115. Email: robertwong123@gmail.com

**Keywords:** Multi-ethnic disparities, Proximal colon cancer, Health care barriers, Cancer screening, Cancer biology

## Abstract

**Background:**

Recent studies have reported on the changing epidemiology of colon cancer. Given this cancer's high prevalence and mortality, defining high risk groups will be important to guide improvements in cancer screening programs.

**Methods:**

A retrospective cohort study of a large population-based cancer registry in the United States from 1973-2004 was performed to analyze the race and sex-specific disparities in colon cancer epidemiology.

**Results:**

Blacks and females demonstrated the greatest proportions of proximal cancers: the incidence rate of proximal cancers among black males was more than double that of Asian males (25.2 per 100,000/year vs 11.7 per 100,000/year, p < 0.0001) and the rate among black females was twice that of Asian females (21.9 per 100,000/year vs 11.4 per 100,000/year, p < 0.0001). Blacks as a group had the highest rates of advanced cancers: the rate among black males was nearly double that of Hispanic males (17.1 per 100,000/year vs 8.7 per 100,000/year, p < 0.0001) and the rate of advanced cancers among black females was twice that of Hispanic females (12.4 per 100,000/year vs 6.2 per 100,000/year, p < 0.0001).

**Conclusions:**

This study demonstrates marked disparities in the sex-specific and race/ethnicity-specific epidemiology of colon cancer. These differences likely represent unequal access to health care resources and race and sex-specific variations in cancer biology. An individualized approach incorporating these disparities would benefit future research and guidelines for improvements in cancer screening programs.

## Introduction

Colon cancer is the second most common cause of death from cancer in the United States [1, 2]. The increasing awareness and implementation of screening programs have led to declines in cancer incidence and mortality [3-8]. However, recent studies have suggested that current screening techniques may be limited in effectively detecting cancers in the entire colon, showing no significant reduction in proximal colon cancer mortality associated with colonoscopy screening [9]. In addition, some studies have suggested that the declines in overall colon cancer incidence are coupled with a shift to more proximal sites of cancer distribution [10, 11].

While declines in colon cancer incidence and mortality are promising, subpopulation analyses have suggested that burden of disease remains disproportionately high for certain demographic groups [12-16]. Whether these differences from barriers to health care access or a combination of genetic and environmental factors resulting in more advanced cancers at presentation is unclear. Knowledge of race/ethnicity-specific patterns in colon cancer epidemiology will help guide future discussions on targeted screening and community educational programs.

In this analysis, race/ethnicity-specific and sex-specific trends in colon cancer incidence and distribution were examined using the National Cancer Institute's Surveillance, Epidemiology, and End Results (SEER) cancer registry. This study focused on the patterns of colon cancer distribution by cancer site and stage during a large inclusive study period. Detailed comparisons of colon cancer incidence between demographic groups were correlated with population data to determine variations in burden of disease.

## Methods

### Data sources

Data were obtained from a population-based cancer registry representing approximately 26% of the U.S population [17]. The 1973-2004 population includes data from 17 regions within the following states: Connecticut, Michigan, Hawaii, Iowa, New Mexico, California, Washington, Utah, Georgia, Alaska, Kentucky, Louisiana, and New Jersey [17, 18]. Incidence data for expanded race classifications were only available from 1992-2004; thus the incidence comparisons were limited to this interval.

### Definitions

Colon cancer cases were identified using the International Classification of Disease for Oncology, 3rd edition [19]. This study utilized SEER's expanded race/ethnicity classifications: non-Hispanic whites, blacks, Asian/Pacific Islanders (Asians), and white Hispanics (Hispanics). Few data from other groups (American Indian/Alaskan Natives, black Hispanics) precluded precise analyses and were not included in this study [18]. SEER also records the anatomic site of cancer distribution. This study used the following site definitions: proximal colon (cecum, appendix, ascending colon, and hepatic flexure), transverse colon, and distal colon (splenic flexure, descending colon, sigmoid colon, rectosigmoid junction, and rectum). Cancer staging definitions were based on SEER staging systems, which are unique to the SEER database and used primarily for describing extent of disease, and not necessarily for prognostic determination [20]. Localized cancers are confined to the colon without transmural invasion to or beyond the serosa. Regional cancers involve spread to regional lymph nodes or direct extension to adjacent tissue. Distant or advanced cancers include metastatic disease and cancers with distant lymph node involvement.

### Statistical analysis

All analyses were performed using the SEER*Stat 6.5.3 program (National Cancer Institute, Maryland) and the Stata statistical package (release ten, Stata Corporation, Texas). P-values for comparisons were calculated with the z-statistic using standard equations [21]. Annual incidence rates are per 100,000 and were age-adjusted to the U.S standard population of 2000. Calendar year-specific rates were calculated based on 3-year and 4-year averages depending on the number of years available for inclusion.

## Results

### Overview

Overall, from 1973-2004, over 500,000 cases of colon cancer were identified. Among all groups, the distal colon was the most common site of cancer involvement, and the majority of cancers were localized staged at diagnosis (Table 1).

**Table 1:** Overall colon cancer distribution by site of cancer and stage of cancer at diagnosis, stratified by race/ethnicity, 1973-2004.

	Distribution by Site					
	Proximal	Percent (%)	Transverse	Percent (%)	Distal	Percent (%)
Non-hispanic white	136139	32.8%	29509	7.1%	249045	60.1%
Black	15459	35.3%	3228	7.4%	25150	57.4%
Asians	7099	23.2%	1802	5.9%	21692	70.9%
Hispanic	7653	29.9%	1384	5.4%	16541	64.7%

	Distribution by Stage					
	Localized	Percent (%)	Regional	Percent (%)	Advanced	Percent (%)
Non-hispanic white	166064	41.4%	155428	38.7%	79993	19.9%
Black	15966	37.8%	15546	36.8%	10732	25.4%
Asians	12315	41.4%	11947	40.1%	5509	18.5%
Hispanic	9811	39.3%	9774	39.2%	5358	21.5%

The percentages are calculated horizontally across each row and represent the proportion of cancers within each race/ethnic group.

### Colon cancer distribution

Table 1 presents the overall colon cancer distribution by anatomic site and cancer stage from 1973-2004. The greatest proportion of distal cancers was seen among Asians (distal, 70.9%; proximal, 23.2%), and the greatest proportion of proximal cancers was seen among blacks (proximal, 35.3%; distal, 57.4%). Among all race/ethnic groups, localized cancers represented about 40% of colon cancers. The proportion of advanced cancers was greatest among blacks (25.4%) and lowest among Asians (18.5%), p < 0.0001.

**Figure 1:**
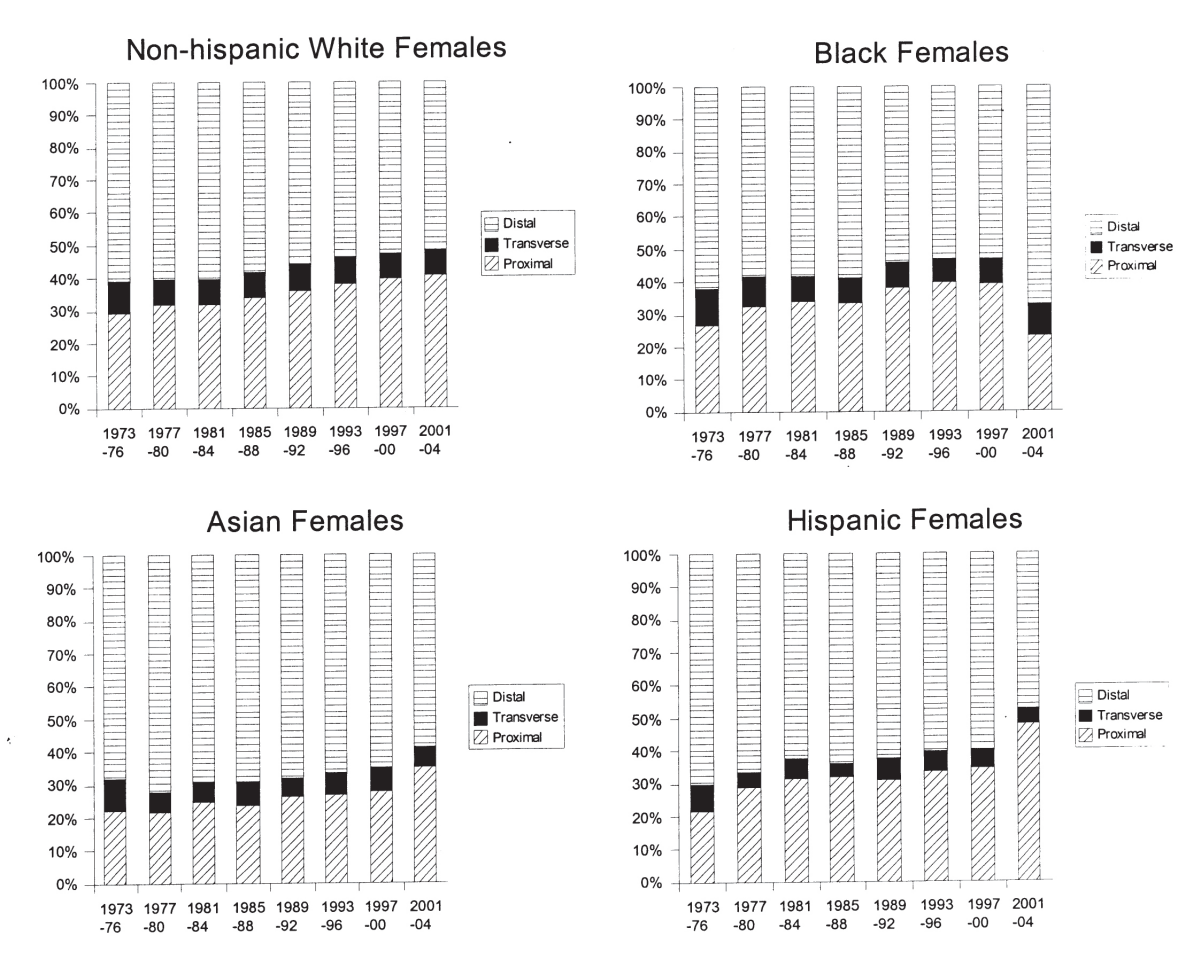
Colon cancer distribution by anatomical site and year, 1973-2004. Males, stratified by race/ethnicity.

**Figure 2:**
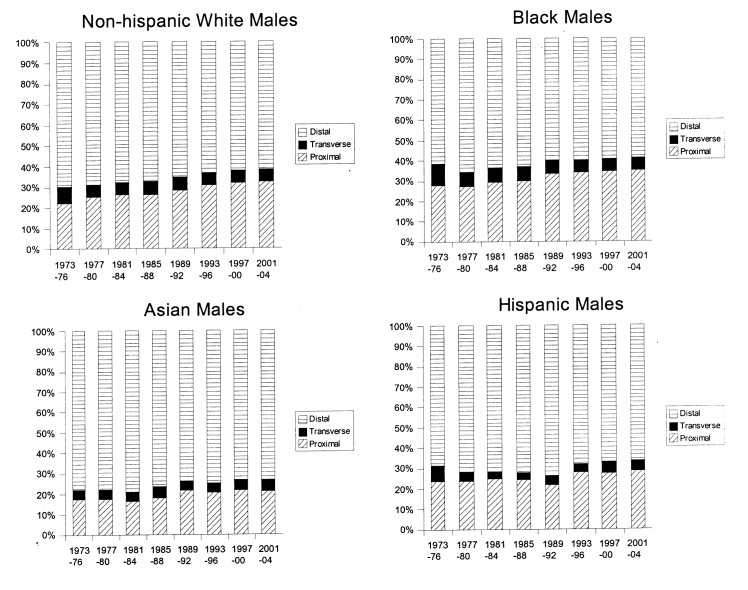
Colon cancer distribution by anatomical site and year, 1973-2004. Females, stratified by race/ethnicity.

Figure 1 and 2 show trends in anatomic distribution of colon cancer by 4-year intervals, stratified by sex and race/ethnicity. Among males, all groups demonstrated a gradual shift towards more proximal distributions of colon cancers. The greatest absolute percentage change was seen among non-Hispanic white males (proximal: 22.4% to 32.5%, p < 0.0001, Fig. 1). Black males also demonstrated a gradual trend towards increased proportion of proximal cancers diagnosed (27.5% to 34.7%, p < 0.0001) and remained the group with the greatest proportion of proximal cancers for all time periods analyzed.

Similar trends towards increased proximal distributions of cancers were seen among females. The greatest absolute increase in the proportion of proximal cancers was seen among Hispanic females (21.5% to 47.7%, p < 0.0001, Fig. 2). Sex-specific comparisons demonstrated greater proportions of proximal cancers among females. For example, for the 2001-2004 time period, proximal distributions represented 28.2% of cancers among Hispanic males and 47.7% of cancers among Hispanic females, p < 0.0001. This sex-specific disparity in proximal cancer distribution was seen among all race/ethnic groups and among the majority of time intervals studied (Fig. 1 and 2).

### Colon cancer incidence by anatomic site and cancer stage

Table 2 shows the average incidence rates of colon cancer from 1992-2004, stratified by sex, race/ethnicity, and anatomic site by 3-year intervals, with the exception of the last interval being a 4-year average because of the odd number of years available for analysis. Among both males and females, blacks had the highest rates of both proximal and distal cancers. For example, during the 2001-2004 interval, the incidence rate of proximal cancers among black males was more than double that of Asian males, the group with the lowest rate (25.2 per 100,000/year vs 11.7 per 100,000/year, p < 0.0001) and the rate of proximal cancers among black females was twice that of Asian females (21.9 per 100,000/year vs 11.4 per 100,000/year, p < 0.0001).

**Table 2:** Average colon cancer incidence rates among males and females per 100,000 population/year, stratified by anatomic site, 1992-2004.

	1992-94		1995-97		1998-2000		2001-04	
	Rate (count)	95% CI	Rate (count)	95% CI	Rate (count)	95% CI	Rate (count)	95% CI
**Males**								
**Total Cancers**								
Non Hispanic white	68.7 (20211)	(67.7-69.6)	65.9 (2098)	(65.0-66.8)	65.4 (20747)	(64.5-66.3)	58.6 (26075)	(57.9-59.3)
Black	75.9 (2232)	(72.6-79.3)	73.2 (2312)	(70.0-76.4)	75.1 (2509)	(72.0-78.2)	72.8 (3589)	(70.3-75.3)
Asians	59.3 (1959)	(56.6-62.1)	58.6 (2252)	(56.2-61.2)	56.4 (2493)	(54.2-58.7)	53.0 (3688)	(51.3-54.8)
Hispanic	48.7 (1456)	(45.8-51.3)	50.0 (1710)	(47.4-52.6)	51.0 (1972)	(48.5-53.5)	46.3 (2976)	(44.5-48.1)
								
**Proximal Cancers**								
Non Hispanic white	20.1 (5872)	(19.6-20.7)	20.4 (6080)	(19.8-20.9)	20.3 (6312)	(19.8-20.8)	19.0 (8295)	(18.6-19.4)
Black	25.1 (722)	(23.2-27.1)	23.7 (734)	(21.9-25.6)	26.8 (856)	(24.9-28.7)	25.2 (1206)	(23.6-26.8)
Asians	12.2 (389)	(10.9-13.5)	12.4 (467)	(11.3-13.6)	12.3 (521)	(11.2-13.4)	11.7 (767)	(10.9-12.6)
Hispanic	13.8 (381)	(12.3-15.4)	13.9 (460)	(12.5-15.3)	14.4 (514)	(13.1-15.9)	13.0 (791)	(12.1-14.0)
								
**Distal Cancers**								
Non Hispanic white	41.7 (12495)	(41.0-42.5)	39.1 (12111)	(38.4-39.8)	38.7 (12446)	(38.0-39.3)	33.8 (15262)	(33.3-34.4)
Black	41.8 (1264)	(39.4-44.3)	41.2 (1337)	(38.9-43.6)	39.1 (1368)	(37.0-41.4)	39.1 (1984)	(37.3-40.9)
Asians	42.5 (1422)	(40.3-44.9)	41.5 (1615)	(39.5-43.6)	40.2 (1801)	(38.3-42.1)	36.7 (2608)	(35.3-38.2)
Hispanic	30.8 (961)	(28.7-33.0)	31.9 (1118)	(29.8-34.0)	32.0 (1280)	(30.1-33.9)	29.1 (1927)	(27.8-30.6)
								
**Females**								
**Total Cancers**								
Non Hispanic white	48.0 (19970)	(47.3-48.7)	47.4 (20167)	(46.7-48.1)	48.0 (20810)	(47.4-48.7)	43.9 (25899)	(43.4-44.5)
Black	57.2 (2460)	(55.0-59.6)	55.9 (2553)	(53.8-58.2)	57.7 (2833)	(55.6-59.8)	54.2 (3879)	(52.5-56.0)
Asians	40.9 (1626)	(38.9-43.0)	38.1 (1801)	(36.3-39.9)	39.2 (2193)	(37.5-40.9)	37.8 (3378)	(36.5-39.1)
Hispanic	33.7 (1358)	(31.9-35.6)	32.4 (1464)	(30.7-34.1)	34.0 (1739)	(32.4-35.7)	31.2 (2582)	(30.0-32.5)
								
**Proximal Cancers**								
Non Hispanic white	17.0 (7201)	(16.6-17.4)	17.0 (7429)	(16.6-17.4)	18.1 (8019)	(17.7-18.5)	17.0 (10267)	(16.7-17.4)
Black	21.1 (900)	(19.7-22.5)	21.7 (986)	(20.4-23.2)	22.3 (1077)	(21.0-23.7)	21.9 (1544)	(20.5-23.1)
Asians	11.2 (424)	(10.2-12.4)	9.6 (440)	(8.7-10.5)	11.1 (605)	(10.2-12.0)	11.4 (999)	(10.7-12.2)
Hispanic	10.9 (423)	(9.9-12.0)	11.0 (474)	(10.0-12.1)	11.9 (586)	(10.9-12.9)	11.5 (906)	(10.7-12.3)
								
**Distal Cancers**								
Non Hispanic white	25.6 (10439)	(25.1-26.1)	24.8 (10301)	(24.3-25.3)	24.7 (10413)	(24.3-25.2)	22.0 (12599)	(21.6-22.4)
Black	29.5 (1279)	(27.9-31.2)	27.7 (1271)	(26.2-29.3)	28.3 (1416)	(26.9-29.9)	25.8 (1878)	(24.6-27.0)
Asians	25.9 (1063)	(24.3-27.3)	24.6 (1189)	(23.2-26.1)	24.1 (1375)	(22.8-25.4)	23.3 (2110)	(22.3-24.3)
Hispanic	19.7 (814)	(18.3-21.1.)	17.8 (842)	(16.6-19.1)	18.7 (990)	(17.6-20.0)	16.6 (1432)	(15.8-17.5)

Annual incidence rates are age-standardized to U.S. population of 2000 and are stratified by sex, year, anatomic site, and race/ethnicity. Proximal refers to cancers involving cecum, appendix, ascending colon, and hepatic flexure. Distal refers to cancers involving splenic flexure, descending colon, sigmoid colon, rectosigmoid junction, and rectum. “Total cancers” include proximal, transverse, and distal sites of the colon.

While all groups showed a trend towards decreased incidence of distal colon cancers, the incidence of proximal cancers remained steady for most groups, and even demonstrated a upward trend in some groups (Table 2). For example, among black males the incidence of distal cancers decreased from 41.8 per 100,000/year to 39.1 per 100,000/year, p = 0.08, whereas the incidence of proximal cancers among the same group was essentially unchanged (25.1 per 100,000/year to 25.2 per 100,000/year, p = 0.94). Among females, rates of distal cancers demonstrated a downward trend in all race/ethnic groups. However, the rates of proximal cancers among females remained the same or increased in all groups (Table 2).

Table 3 shows the average incidence rates of colon cancer from 1992-2004, stratified by sex, race/ethnicity, and cancer stage using the same time intervals. Among all race/ethnic groups, blacks had one of the highest incidence rates of cancer for all stages. Race/ethnicity-specific comparisons demonstrated significant disparities. For example, during 2001-2004, the rate of advanced cancers among black males was nearly double that of Hispanic males (17.1 per 100,000/year vs 8.7 per 100,000/year, p < 0.0001) and the rate of advanced cancers among black females was twice that of Hispanic females (12.4 per 100,000/year vs 6.2 per 100,000/year, p < 0.0001). Race/ethnicity specific disparities among localized cancers were not as robust. For example, from 2001-2004, the rate of localized cancers among black males was 50% greater than Hispanic males (26.3 per 100,000/year vs 17.6 per 100,000/year, p < 0.0001) and the rate among black females was 60% greater than among Hispanic females (18.3 per 100,000/year vs 12.1 per 100,000/year, p < 0.0001).

**Table 3:** Average colon cancer incidence rates among males and females per 100,000 population/year, stratified by cancer stage, 1992-2004.

	1992-94		1995-97		1998-2000		2001-04	
	Rate (count)	95% CI	Rate (count)	95% CI	Rate (count)	95% CI	Rate (count)	95% CI
**Males**								
**Localized Cancers**								
Non-hispanic white	27.1 (7992)	(26.5-27.7)	25.8 (7888)	(25.2-26.4)	26.9 (8585)	(26.4-27.5)	25.1 (11184)	(24.7-25.6)
Black	26.8 (776)	(24.9-28.9)	26.4 (843)	(24.5-28.3)	26.8 (890)	(25.0-28.7)	26.3 (1292)	(24.8-27.9)
Asians	23.9 (787)	(22.2-25.7)	23.9 (922)	(22.3-25.5)	22.9 (1018)	(21.5-24.4)	22.0 (1526)	(20.9-23.1)
Hispanic	16.9 (516)	(15.3-18.5)	19.5 (659)	(17.9-21.2)	18.8 (725)	(17.3-20.4)	17.6 (1116)	(16.5-18.7)
								
**Regional Cancers**								
Non-hispanic white	24.3 (7229)	(23.8-24.9)	23.5 (7198)	(22.9-24.0)	23.6 (7512)	(23.1-24.2)	20.5 (9114)	(20.0-20.9)
Black	25.1 (770)	(23.3-27.1)	23.4 (747)	(21.7-25.3)	25.2 (855)	(23.5-27.1)	24.0 (1202)	(22.6-25.5)
Asians	21.7 (728)	(20.1-23.4)	21.6 (833)	(20.1-23.1)	21.5 (956)	(20.1-22.9)	18.8 (1315)	(17.8-19.8)
Hispanic	18.5 (546)	(16.8-20.2)	17.7 (614)	(16.2-19.3)	18.2 (730)	(16.8-19.7)	17.1 (1105)	(16.0-18.2)
								
**Advanced Cancers**								
Non-hispanic white	12.7 (3786)	(12.3-13.1)	12.3 (3814)	(11.9-12.7)	11.4 (3657)	(11.0-11.8)	10.4 (4685)	(10.1-10.7)
Black	16.7 (506)	(15.2-18.3)	16.6 (528)	(15.1-18.1)	17.5 (594)	(16.1-19.1)	17.1 (873)	(15.9-18.3)
Asians	10.1 (344)	(9.0-11.3)	9.7 (377)	(8.7-10.7)	9.2 (415)	(8.4-10.2)	10.0 (706)	(9.3-10.8)
Hispanic	10.1 (306)	(8.9-11.4)	9.6 (349)	(8.5-10.8)	10.1 (398)	(9.0-11.2)	8.7 (598)	(7.9-9.5)
								
**Females**								
**Localized Cancers**								
Non-hispanic white	17.9 (7440)	(17.5-18.3)	17.9 (7576)	(17.5-18.5)	19.2 (8269)	(18.8-19.6)	18.3 (10700)	(17.9-18.6)
Black	19.8 (849)	(18.5-21.2)	20.0 (908)	(18.7-21.4)	20.9 (633)	(19.6-22.2)	19.2 (1366)	(18.1-20.2)
Asians	14.7 (601)	(13.5-16.0)	15.5 (734)	(14.3-16.6)	14.9 (839)	(13.9-15.9)	14.8 (1328)	(14.0-15.6)
Hispanic	11.2 (455)	(10.2-12.3)	11.3 (503)	(10.3-12.4)	12.4 (1024)	(11.4-13.4)	12.1 (993)	(11.3-12.8)
								
**Regional Cancers**								
Non-hispanic white	18.1 (7495)	(17.7-18.6)	17.7 (7524)	(17.3-18.2)	18.1 (7780)	(17.6-18.5)	15.6 (9180)	(15.3-16.0)
Black	19.5 (842)	(18.2-20.9)	19.4 (892)	(18.8-20.7)	19.3 (948)	(18.1-20.6)	18.9 (1347)	(17.9-19.9)
Asians	16.5 (654)	(15.2-17.8)	14.1 (672)	(13.1-15.3)	15.8 (884)	(14.7-16.8)	15.0 (1343)	(14.2-15.8)
Hispanic	13.4 (546)	(12.3-14.6)	12.3 (563)	(11.2-13.3)	12.9 (663)	(11.9-13.9)	10.8 (900)	(10.1-11.5)
								
**Advanced Cancers**								
Non-hispanic white	8.9 (3646)	(8.6-9.1)	8.8 (3672)	(8.5-9.0)	8.1 (3448)	(7.8-8.4)	7.8 (4538)	(7.6-8.0)
Black	12.5 (552)	(11.4-13.6)	11.9 (549)	(10.9-12.9)	12.8 (639)	(11.8-13.8)	12.4 (903)	(11.6-13.3)
Asians	6.3 (257)	(5.5-7.1)	5.9 (292)	(5.3-6.7)	6.6 (376)	(6.0-7.3)	6.5 (588)	(6.0-7.1)
Hispanic	6.7 (272)	(5.9-7.6)	6.8 (318)	(6.1-7.7)	6.7 (353)	(6.0-7.4)	6.2 (524)	(5.6-6.7)

Annual incidence rates are age-standardized to U.S. population of 2000 and are stratified by sex, year, cancer stage, and race/ethnicity. Localized stages refer to cancers confined to the colon without transmural invasion to or beyond the serosa. Regional stages involve spread to regional lymph nodes or direct extension to adjacent tissue. Advanced cancers include metastatic disease and cancers with distant lymph node involvement.

While cancer rates among non-Hispanic whites and Asians showed a downward trend for all stages, rates among blacks and Hispanics were unchanged. From 1992-1994 to 2001-2004, the rates of localized cancers among Hispanic males (16.9 per 100,000/year to 17.6 per 100,000/year, p = 0.47) and the rates of advanced cancers among black males (16.7 per 100,000/year to 17.1 per 100,000/year, p = 0.69) did not show significant changes (Table 3).

### Colon cancer incidence using combined stage/site comparisons

Tables 2 and 3 demonstrate that blacks have the highest cancer rates of proximal and advanced cancers. A combined cancer stage/site comparison may help delineate the etiology of these disparities. Table 4 shows the overall average incidence rates of colon cancer from 1992-2004 using a combined cancer stage and anatomic site comparison, stratified by sex and race/ethnicity. Among males and females, blacks had the greatest incidence rate of proximal cancers among both localized and advanced stages. For example, the rate of advanced proximal cancers among black males was more than double that of Asian males (6.0 per 100,000/year vs 2.5 per 100,000/year, p < 0.001) and the rate of advanced proximal cancers among black females was more than double that of Asian females (5.2 per 100,000/year vs 2.0 per 100,000/year, p < 0.001). Among distal cancers, the sex-specific disparities were not as robust. For example, the incidence rate of advanced distal cancers among black males was 50% greater than that of Hispanic males (8.5 per 100,000/year vs 5.6 per 100,000/year, p < 0.001) and the rate of advanced distal cancers among black females was 70% greater than that of Hispanic females (5.4 per 100,000/year vs 3.1 per 100,000/year, p < 0.001).

**Table 4:** Average colon cancer incidence rates among males and females per 100,000 population/year, stratified by cancer stage and anatomic site, 1992-2004.

	Localized cancers			Advanced cancers		
	Rate (count)	95% CI	% of all cancers	Rate (count)	95% CI	% of all cancers
**Males**						
**Proximal cancers**						
Non-Hispanic white	7.4 (9763)	(7.2-7.5)	38.1%	3.9 (5274)	(3.8-4.0)	20.6%
Black	8.6 (1167)	(8.1-9.1)	34.5%	6.0 (871)	(3.6-6.5)	25.7%
Asians	4.4 (773)	(4.1-4.7)	37.1%	2.5 (443)	(2.2-2.7)	21.5%
Hispanic	4.6 (717)	(4.3-4.9	34.7%	2.7 (448)	(2.5-3.0)	21.7%
						
**Distal cancers**						
Non-Hispanic white	17.0 (23515)	(16.8-17.2)	47.1%	6.2 (8610)	(6.0-6.3)	17.2%
Black	15.9 (2365)	(15.2-16.6)	42.6%	8.5 (1290)	(8.0-9.0)	23.2%
Asians	17.2 (3236)	(16.6-17.8)	45.3%	6.2 (1196)	(5.8-6.6)	16.8%
Hispanic	12.5 (2137)	(11.9-13.1)	42.5%	5.6 (1020)	(5.3-6.0)	20.3%
						
**Females**						
**Proximal cancers**						
Non-Hispanic white	6.3 (12128)	(6.2-6.4)	38.2%	3.3 (6119)	(3.2-3.4)	19.3%
Black	7.3 (1492)	(6.9-7.7)	34.8%	5.2 (1107)	(4.9-5.6)	25.8%
Asians	3.7 (835)	(3.5-4.0)	35.1%	2.0 (469)	(1.8-2.2)	19.7%
Hispanic	3.9 (810)	(3.6-4.2)	35.1%	2.4 (522)	(2.2-2.6)	22.6%
						
**Distal cancers**						
Non-Hispanic white	10.7 (19281)	(10.5-10.8)	46.5%	3.9 (6953)	(3.8-4.0)	16.8%
Black	11.0 (2336)	(10.6-11.5)	43.0%	5.4 (1152)	(5.1-5.7)	21.2%
Asians	10.2 (2430)	(9.8-10.6)	29.6%	3.7 (889)	(3.5-3.9)	16.2%
Hispanic	7.2 (1623)	(6.8-7.5)	42.0%	3.1 (735)	(2.9-3.4)	19.0%

Annual incidence rates are age-standardized to U.S. population of 2000 and are stratified by sex, cancer stage, anatomic site, and race/ethnicity. Percentages are calculated horizontally across each row and represent the proportion of cancers within each race/ethnic group. The proportion of regional staged cancers are not presented in this table.

## Discussion

Colon cancer is one of the leading causes of cancer death in the United States [1, 2]. Advances in cancer prevention and screening programs have lead to dramatic declines in overall cancer incidence and improvements in cancer survival [3-8]. The success of colon cancer screening programs can be evaluated by its effects on cancer incidence, survival, and the stage/severity of cancers detected. Recent studies have suggested that colonoscopy screening does not reduce mortality from proximal cancers [9]. Identifying specific cohorts with an increased trend towards proximal distribution of cancers is a first step in targeting disease-specific disparities.

In this study, a steady trend towards an increased proximal distribution of cancers was seen among all groups (Fig. 1 and 2). Sex-specific and race/ethnicity-specific comparisons demonstrated that females and blacks had the greatest proportion of proximal cancers (Tables 1 and 2). As a result, blacks and females as a group would benefit most from complete rather than limited screening examinations (e.g. sigmoidoscopy). Furthermore, these data suggest that any targeted approach to address the lack of a survival benefit from current colon cancer screening programs for proximally located cancers should focus on blacks and females.

This study demonstrated striking disparities in sex-specific and race/ethnicity specific incidence rates of colon cancer. Cancer incidence among blacks was one of the highest among both males and females and among all cancer sites and stages (Tables 2- 4). Whether these race/ethnicity-specific disparities represent variations in cancer biology or unequal access to health care resources is unclear. A detailed analysis using a combined cancer stage/cancer site comparison may help delineate some of the underlying factors. Current colon cancer screening programs are associated with improved survival from left-sided or distal cancers [3-9]. The association between cancer screening and right-sided or proximal cancers is less clear [9]. As a result, focusing specifically on the epidemiology of distal cancers may contribute information regarding the etiology of race/ethnicity-specific disparities in cancer incidence. Identifying more advanced distal cancers among certain groups would suggest impaired access to cancer screening programs and not necessarily cancer biology because standard screening examinations would have identified these cancers earlier. In this study, blacks as a group had significantly greater rates of advanced distal cancers. The rates of localized distal cancers were also significantly greater among blacks. The greater rates of advanced distal cancers among blacks likely represent delayed cancer screening, possibly a result of barriers or limitations to timely cancer screening. It is conceivable that genetics and cancer biology may also be different among blacks leading to more aggressive disease at presentation. However, if this were the case, the increased rates of advanced cancers would be seen concurrently with relatively lower rates of localized cancers. In this study, higher cancer incidence was seen for blacks among both localized and advanced stages of distal cancers (Table 4). Limitations in health care access along with race/ethnicity-specific variations in cancer incidence are likely to play important roles in the cancer disparities seen. A targeted cancer screening approach that takes into account the race/ethnicity-specific and sex-specific variations in colon cancer incidence will likely have the greatest benefit.

Disparities among proximal colon cancers is less clear in its implications given recent findings showing no survival benefit from colonoscopy for right-sided or proximal colon cancers [9]. While certain groups being offered alternative screening tests (fecal occult blood, sigmoidoscopy) with poorer sensitivity for detecting proximal tumors may account for the greater proportion of proximal cancers, the study by Baxter, et al. included patients that were all exposed to complete colonoscopy [9]. Current screening techniques may lack sensitivity for detecting proximal colon cancers, leading to more advanced disease at presentation. As a result, sex-specific and race/ethnicity-specific disparities in the incidence of proximal cancers may not necessarily represent variations in access to health care. Identifying a greater incidence of advanced proximal cancers may represent variations in cancer biology. The fact that current screening programs reduce mortality for proximal cancers, and thereby do not affect the natural progression of these cancers suggest that sex-specific or race/ethnicity-specific variations in disease severity among proximal cancers may more likely result from variations in cancer biology. In this study, blacks demonstrated significantly higher rates of advanced proximal cancers among males and females. This finding may represent genetic or biological variations in this group that are race/ethnicity-specific.

While it is known that colon cancer rates are higher and tend to be more aggressive among blacks, unequal access to health care is also well documented among this group [13, 22-27]. The possibility that minority groups have suboptimal access to cancer education and health care resources is undeniable and further interventions to target these under-represented groups are needed. Community programs targeting minority groups and greater awareness among health care professionals should be a major component of any cancer prevention and screening intervention [27, 28]. Furthermore, an individualized approach to research and improvements in colon cancer screening guidelines would benefit from incorporating the sex-specific and race/ethnicity-specific disparities observed in this study.

The strengths of this study include the utilization of high quality data from a population-based cancer registry that represents a large proportion of the United States and is globally recognized as an authoritative source of information on cancer incidence and survival in the U.S. [17, 18]. A large inclusive study period with detailed data for expanded race/ethnic groups permitted accurate assessment of epidemiological data that is generalizable to the U.S. population.

Our analysis is limited by factors inherent in registry data. The lack of available data on types of colon cancer screening techniques used prevented the analysis of associations between various screening examinations and the disparities observed. The SEER staging system used in this study is not the most effective staging system used in clinical practice and the simplicity of this staging system does not account for the spectrum of disease that exists within each category. However, even if this were the case, it would not fully account for the gender-specific and race/ethnicity-specific disparities this study demonstrates. For the sake of consistency and accuracy of comparisons across all groups and time periods, our study utilized the SEER staging system.

In conclusion, our study describes marked sex-specific and race/ethnicity-specific disparities in colon cancer epidemiology. Significantly higher rates of advanced cancers among blacks and proximal cancers among females may represent disparities in health care access or sex-specific and race/ethnicity-specific variations in cancer biology. Any future attempts to improve cancer prevention and screening programs should focus on interventions to address the disparities seen among these groups. Further research and programs to improve cancer screening would benefit from a targeted approach taking into account these sex-specific and race/ethnicity-specific cancer disparities. More studies are needed to clarify the etiology behind these variations in cancer epidemiology and to determine what effect this has on overall colon cancer survival.

## References

[1] Parkin DM, Bray F, Ferlay J, Pisani P (2005). Global cancer statistics, 2002. CA Cancer J Clin.

[2] U.S. Cancer Statistics Working Group (2007). United States Cancer Statistics: 2004 Incidence and Mortality.

[3] Mandel JS, Bond JH, Church TR, Snover DC, Bradley GM, Schuman LM, Ederer F (1993). Reducing mortality from colorectal cancer by screening for fecal occult blood. Minnesota Colon Cancer Control Study. N Engl J Med.

[4] Mandel JS, Church TR, Bond JH, Ederer F, Geisser MS, Mongin SJ, Snover DC (2000). The effect of fecal occult-blood screening on the incidence of colorectal cancer. N Engl J Med.

[5] Selby JV, Friedman GD, Quesenberry CP, Weiss NS (1992). A case-control study of screening sigmoidoscopy and mortality from colorectal cancer. N Engl J Med.

[6] Muller AD, Sonnenberg A (1995). Prevention of colorectal cancer by flexible endoscopy and polypectomy. A case-control study of 32,702 veterans. Ann Intern Med.

[7] Winawer SJ, Zauber AG, Ho MN, O'Brien MJ, Gottlieb LS, Sternberg SS, Waye JD (1993). Prevention of colorectal cancer by colonoscopic polypectomy. The National Polyp Study Workgroup. N Engl J Med.

[8] Cooper GS, Yuan Z, Landefeld CS, Johanson JF, Rimm AA (1995). A national population-based study of incidence of colorectal cancer and age. Implications for screening in older Americans. Cancer.

[9] Baxter NN, Goldwasser MA, Paszat LF, Saskin R, Urbach DR, Rabeneck L (2009). Association of colonoscopy and death from colorectal cancer. Ann Intern Med.

[10] Rabeneck L, Davila JA, El-Serag HB (2003). Is there a true "shift" to the right colon in the incidence of colorectal cancer?. Am J Gastroenterol.

[11] Cucino C, Buchner AM, Sonnenberg A (2002). Continued rightward shift of colorectal cancer. Dis Colon Rectum.

[12] Ward E, Jemal A, Cokkinides V, Singh GK, Cardinez C, Ghafoor A, Thun M (2004). Cancer disparities by race/ethnicity and socioeconomic status. CA Cancer J Clin.

[13] Agrawal S, Bhupinderjit A, Bhutani MS, Boardman L, Nguyen C, Romero Y, Srinivasan R (2005). Colorectal cancer in African Americans. Am J Gastroenterol.

[14] Chu KC, Miller BA, Springfield SA (2007). Measures of racial/ethnic health disparities in cancer mortality rates and the influence of socioeconomic status. J Natl Med Assoc.

[15] Irby K, Anderson WF, Henson DE, Devesa SS (2006). Emerging and widening colorectal carcinoma disparities between Blacks and Whites in the United States (1975-2002). Cancer Epidemiol Biomarkers Prev.

[16] Kauh J, Brawley OW, Berger M (2007). Racial disparities in colorectal cancer. Curr Probl Cancer.

[17] Ries LAG, Melbert D, Krapcho M (2007). SEER Cancer Statistics Review, 1975-2004.

[18] Surveillance, Epidemiology, and End Results (SEER) Program Public Use CD-ROM (1973-2004). National Cancer Instutite, DCCPS, Surveillance Research Program, Cancer Statistics Branch, released April 2007

[19] World Health Organization (2000). International Classification of Diseases for Oncology.

[20] Young JL, Roffers SD, Ries LAG (2001). SEER Summary Staging Manual – 2000: Codes and Coding Instructions.

[21] Silva IS (2001). Cancer Epidemiology: Principles and Methods.

[22] Fiscella K, Franks P, Gold MR, Clancy CM (2000). Inequality in quality: addressing socioeconomic, racial, and ethnic disparities in health care. JAMA.

[23] Brown ER, HJKF Foundation Racial and Ethnic Disparities in Access to Health Insurance and Health Care.

[24] Schneider EC, Leape LL, Weissman JS, Piana RN, Gatsonis C, Epstein AM (2001). Racial differences in cardiac revascularization rates: does "overuse" explain higher rates among white patients?. Ann Intern Med.

[25] Epstein AM, Weissman JS, Schneider EC, Gatsonis C, Leape LL, Piana RN (2003). Race and gender disparities in rates of cardiac revascularization: do they reflect appropriate use of procedures or problems in quality of care?. Med Care.

[26] Chen VW, Fenoglio-Preiser CM, Wu XC, Coates RJ, Reynolds P, Wickerham DL, Andrews P (1997). Aggressiveness of colon carcinoma in blacks and whites. National Cancer Institute Black/White Cancer Survival Study Group. Cancer Epidemiol Biomarkers Prev.

[27] Carethers JM (1999). Racial and ethnic factors in the genetic pathogenesis of colorectal cancer. J Assoc Acad Minor Phys.

[28] Freeman HP (1998). The meaning of race in science—considerations for cancer research: concerns of special populations in the National Cancer Program. Cancer.

